# A highly polymorphic insertion in the Y-chromosome amelogenin gene can be used for evolutionary biology, population genetics and sexing in *Cetacea *and *Artiodactyla*

**DOI:** 10.1186/1471-2156-9-64

**Published:** 2008-10-16

**Authors:** Matthias Macé, Brigitte Crouau-Roy

**Affiliations:** 1 UMR 5174 UPS/CNRS EDB "Evolution et Diversité biologique", Bât 4R3b2, Université Paul Sabatier, 118 route de Narbonne, 31062 TOULOUSE cedex 9, France; 2 Centre de Physiopathologie de Toulouse Purpan, INSERM U563, CHU Purpan, F-31300 Toulouse, France

## Abstract

**Background:**

The early radiation of the *Cetartiodactyla *is complex, and unambiguous molecular characters are needed to clarify the positions of hippotamuses, camels and pigs relative to the remaining taxa (*Cetacea *and *Ruminantia*). There is also a need for informative genealogic markers for Y-chromosome population genetics as well as a sexing method applicable to all species from this group. We therefore studied the sequence variation of a partial sequence of the evolutionary conserved amelogenin gene to assess its potential use in each of these fields.

**Results and discussion:**

We report a large interstitial insertion in the Y amelogenin locus in most of the *Cetartiodactyla *lineages (cetaceans and ruminants). This sex-linked size polymorphism is the result of a 460–465 bp inserted element in intron 4 of the amelogenin gene of Ruminants and Cetaceans. Therefore, this polymorphism can easily be used in a sexing assay for these species.

When taking into account this shared character in addition to nucleotide sequence, gene genealogy follows sex-chromosome divergence in *Cetartiodactyla *whereas it is more congruent with zoological history when ignoring these characters. This could be related to a loss of homology between chromosomal copies given the old age of the insertion.

The 1 kbp *Amel-Y *amplified fragment is also characterized by high nucleotide diversity (64 polymorphic sites spanning over 1 kbp in seven haplotypes) which is greater than for other Y-chromosome sequence markers studied so far but less than the mitochondrial control region.

**Conclusion:**

The gender-dependent polymorphism we have identified is relevant not only for phylogenic inference within the *Cetartiodactyla *but also for Y-chromosome based population genetics and gender determination in cetaceans and ruminants. One single protocol can therefore be used for studies in population and evolutionary genetics, reproductive biotechnologies, and forensic science.

## Background

About 240 to 320 million years ago, shortly after the divergence of mammalian and avian lineages, progressive X-Y differentiation began, following chromosomal interstitial rearrangements [1]. This resulted in a partial loss of homology between both chromosomes which reached its maximal extent in primates [2]. Amelogenin is the enamel matrix protein that combines with hydroxyapatite crystals to form enamel prisms in teeth [3]. The gene encoding the amelogenin protein (*Amel*) is among the few genes expressed from both X and Y chromosomes in placental mammals (*Eutheria*) [4].

### Evolutionary uncertainties about the basal diversification of *Cetartiodactyla*

The *Cetartiodactyla *(even-toed ungulates, whales and dolphins) radiated approximately 70–80 Myrs ago. The relative positions of the camelid, suiform (pigs), hippopotamus, ruminant and cetacean (whales and dolphins) groups remain unclear, whether morphological or molecular characters are used for attribution [5-9]. Moreover, polytomies (unresolved tree nodes) within some *Cetartiodactyla *taxa [8] highlight areas for further data collection (both species and markers) and phylogenetic research. This is a particularly delicate problem within cetaceans, probably due to adaptative radiations within a short period of time [10,11].

### Y-chromosome sequence markers are needed for population genetics

Males are the heterogametic sex in mammals, and usually, unequal numbers of males and females transmit genes from one generation to the next. Y-specific polymorphisms should allow the inference of sex-specific population parameters and decryption of breeding system patterns and dispersal strategies. Overall, the use of Y-specific markers has been restricted to evolutionary studies of human history and some scarce studies in population genetics, perhaps because of the low diversity of these markers [12]. Within *Cetartiodactyla*, genetic structure or admixture, *e.g*. in sheep [13] or cattle [14,15], has made use of a few Y-specific markers including microsatellites, SNPs and indels.

The matrilineally transmitted mitochondrial control region is commonly used as an informative sequence for population genetics. An equivalent had not been found to date on the Y chromosome. We considered the well-known amelogenin gene to be of particular interest because parts of it do not recombine between X and Y chromosomes.

### Molecular sexing

Sex-chromosome recombination discrepancies have been exploited to develop many molecular sexing techniques. Although it can be ambiguous in some small populations, the amelogenin locus is the most commonly used for gender determination in humans [16]. Accurate gender determination in mammals is crucial to various applications in reproductive technologies, forensic investigations and population management. Some techniques rely on specific amplification of loci localized on the Y chromosome (such as Sry [17]) while others are based on amplification of homologous fragments from both X and Y chromosomes (e.g. ZFX/ZFY [18]) or use both markers [19]. Each of these has limitations, such as the need for multiplexing with other markers or additional steps such as digestion, labelling or sequencing. Several amelogenin-based techniques have expanded the taxonomic coverage of molecular sexing for *Artiodactyla *[20-22] but they have not yet been extended to *Cetacea*. Therefore, there is a need both for new methods that apply to a greater number of species and to increase the number of cross-checking sexing methods, especially in conservation biology [23].

In this study, we found out that sequence variations in the amelogenin locus can be applied in evolutionary and population genetics as well as for molecular sexing in the highly diversified *Cetartiodactyla *group. We therefore carried out an evolutionary study of orthologous *Amel-Y *and *Amel*-*X *sequences (exons 4 to 5) in *Cetartiodactyla*. We studied four *Cetacea *(the striped dolphin, the bottlenosed dolphin, Risso's dolphin and the fin whale) and three *Artiodactyla *(cow, pig and sheep) species.

## Results and Discussion

### Amelogenin can be used for molecular sexing and evolutionary genetics in *Cetartiodactyla*

**Figure 1:**
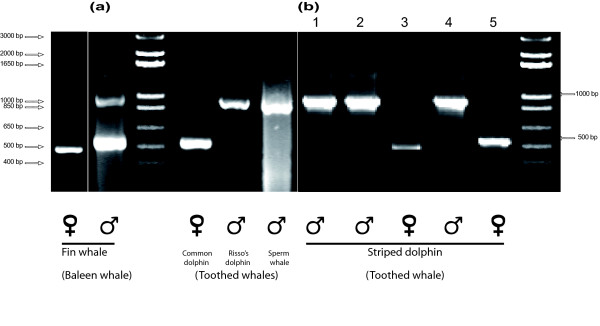
**Sex-related size polymorphism of amelogenin fragment in *Cetacean***. (Molecular weight markers is Biolabs' 1 kb + ladder): a) Agarose gel showing differences between male amplification in a Baleen (toothless) whale (left of the ladder) and Toothed whales (on the right). b) Agarose gel showing differences between males and females in Striped dolphin. 1,000 bp band for *Amel-Y*, 500 bp band for *Amel-X*. Each lane represents a single sample (#1 to 5). Symbols ♂ and ♀ are for male and female samples respectively.

**Figure 2:**
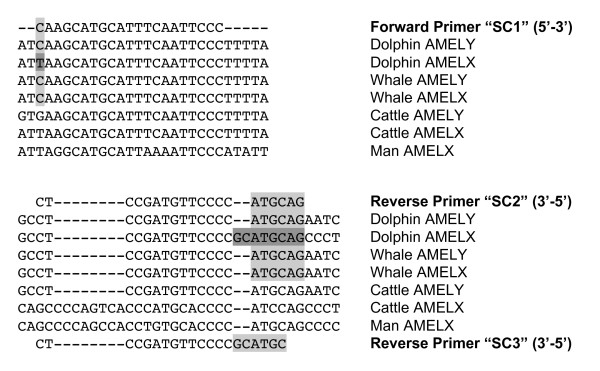
**Sequence alignment of the oligonucleotide primers with target sequences in *Cetacea*, Cattle and Man**. Species and chromosomal location are given on the right side. Shaded columns represents the nucleotide mutated in Dolphins. Accession numbers of sequences follow: Dolphins (EMBL:AM744958–AM744964, EMBL:AM744970–AM744971, EMBL:AM744968, EMBL:AY787743S2 – Y and EMBL:AM744965 – X) and Whales (EMBL:AM744967, EMBL:AM744969 -X- and EMBL:AM744966 – Y), Cattle (GenBank:AB091789 -X- and GenBank:AB091790 – Y) and Man (GenBank:NT_011757 -X- from 9098117 to 9098612 and GenBank:NC_000024 -Y- from 6796200 to 6796719).

Amplification of the studied segment of the amelogenin locus using the species-specific SC1-SC2 primers resulted in an obvious sex-related size polymorphism in all *Cetacea *(Fig. 1) with a unique 521 bp band for females (two *Amel-X *copies) and an additional 980 bp band for the *Amel-Y *in males. This pattern was obvious in male Baleen whales (Mysticetes) but there was no corresponding *Amel-X *amplification in male dolphins unless by using the primers X5-X6 derived from the human amelogenin sequence. Previous studies showed that amelogenin amplification was prone to allelic drop-out or at least to preferential amplification [24]. These phenomena may be explained by several factors. Usually, amplification of the lesser sized allele is favoured when the amount of polymerase is a limiting factor or in case of template DNA degradation [25]. Small amounts o DNA may also increase stochasticity of the annealing [26]. However, our results are not consistent with these situations since the allele favoured (*Amel-Y*) is always the greatest one. On the other hand, differences in GC content and mismatches in the annealing sequences may account for differential amplification. The amelogenin fragments that we studied are characterized by a higher GC content when amplified from the X chromosome (56%) than from Y-chromosome (47%). This difference may result from a non-insertion in the *Amel-X *fragment. This feature as well as a 2 bp-long mismatch between dolphin's *Amel-X *and the 5' end of the reverse primer SC2 (Fig. 2) may favour preferential amplification of the Y copy in dolphins (Fig. 1b). Indeed, amplifying male dolphin samples SC3 (primer without mismatch, see Fig. 2) instead of the SC2, restores the two bands, seen in baleen whales. The presence of this large insertion in the *Amel-Y *copy can be used for sex determination in probably all cetacean species.

**Figure 3:**
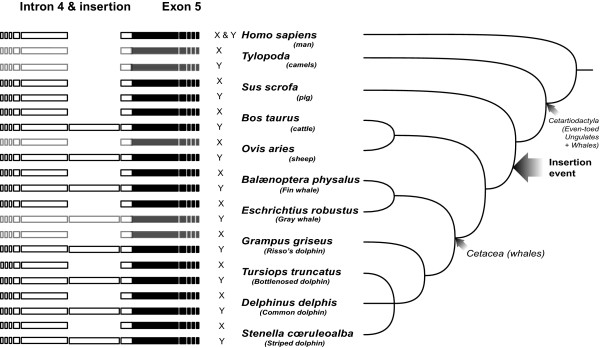
**Schematic representation of the sex-related polymorphism of the amelogenin locus in an evolutionary perspective**. Insertion and intron 4 are represented by a white bar, whereas exon 5 is in black. Shaded bars stands for absent data, deduced from evolutionary relationships. The vertical order links to the "tree of life" view (according [27] among others) provided on the right.

In order to define the breakpoints of the Y insertion location and investigate its evolutionary history, we sequenced various cetaceans (listed in Methods; sequences deposited under the following accessions: EMBL:AM744958 to AM744971). After alignment with available sequences from *Artiodactyla *(see list in Methods), we detected the same polymorphism in all other *Cetartiodactyla *except the Pig (Fig. 3): a 460–465 bp insertion (size is a function of indels within different individuals or species) located between the 4^th ^and 5^th ^exons (188^th ^to 651^st ^position of Y sequences e.g. EMBL:AM744958). Haplotype names and their corresponding accessions are given in Table 1. Sequence similarity was checked by running BLAST (Basic Local Alignment Search Tool) over GenBank nr/nt nucleotide collection sequences with megablast algorithm (intended for high similarity sequences). In addition to the bovine and ovine *Amel-Y*, the only two relevant (78 and 83% homology, E-values 4.10^-68 ^and 3.10^-53^) hits matched a fragment on the seventh chromosome in pigs (ca. 250 bp), suggesting the insertion might be a transposable element.

**Table 1:** List of *Amel-X *and *Amel-Y *haplotype names in *Cetaceans *and their EMBL accession numbers

**Haplotype Name**	**EMBL Accession**
*Stenella cœruleoalba YA1*	AM744963

*Stenella cœruleoalba YA2*	AM744964

*Stenella cœruleoalba YB1*	AM744958

*Stenella cœruleoalba YB2*	AM744959

*Stenella cœruleoalba YB3*	AM744960

*Stenella cœruleoalba YB4*	AM744961

*Stenella cœruleoalba YB5*	AM744962

*Delphinus delphis Y1*	AM744970

*Delphinus delphis Y2*	AM744971

*Stenella cœruleoalba X*	AM744965

*Grampus griseus Y*	AM744968

*Balænoptera physalus Y*	AM744966

*Balænoptera physalus X*	AM744967

*Eschrichtius robustus X*	AM744969

*Tursiops truncatus X*	AY787743S2

**Figure 4:**
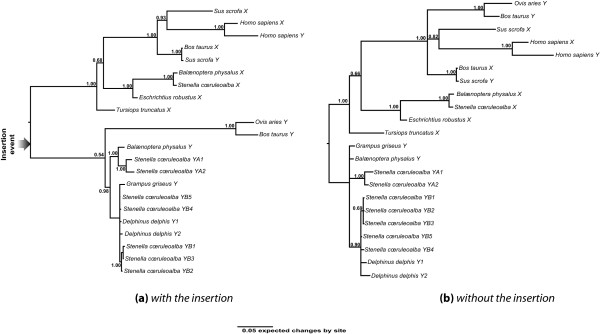
**Comparison of phylogenic trees of the *Amel-X *and *Amel-Y *fragments inferred (a) with the insertion (b) without the insertion**. (a) The phylogenic tree of the complete fragment shows trans-specific clustering by sex chromosome in *Cetartiodactyla*. Tip labels are haplotypes as deposited in the EMBL database; Y and X are for *Amel-Y *and *Amel-X *haplotypes respectively. *Stenella cœruleoalba *haplotypes were named according to population origin (YA/Group 1, YB/Group 2, see Methods). (b) The inferred phylogeny after removing the insertion gives a slightly different picture: trans-specific clustering by sex-chromosome is lost except in *Cetaceans*.

**Figure 5:**
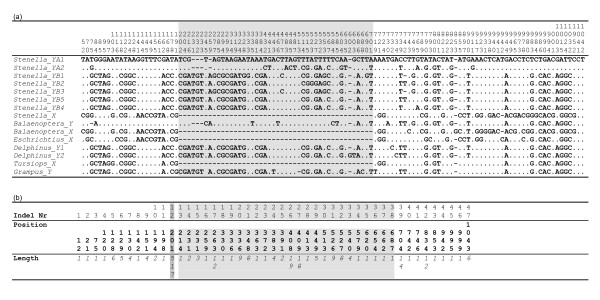
**Polymorphic sites and indels in the *Amel-X *and *Amel-Y *regions in the Cetacean species studied**. (a) Nucleotide positions are represented above and on the left side the names of the haplotypes. All positions are represented on the first sequence and each matching nucleotide on the other haplotypes is represented by a dot. (b) Indels are numbered (first row) following their order on the aligned sequences. They are characterized by their position (second row) and their length (third row). In both tables, shaded areas corresponds to the region holding the large insertion.

We interpret the presence of this insertion as a synapomorphy (shared character) of the *Cetartiodactyla *excluding pigs and probably other early derived groups (camels, hippopotamuses; [27], see Fig. 3). In addition to this long insertion, 46 other indels were detected by sequence alignment (positions and sizes detailed in Figure 5). Indels are particularly useful for testing phylogenic hypotheses, as they can provide information about ancient divergences rather than population information. We therefore assessed whether phylogenetic topologies differed if we took into account or not the information contained in these indels. Thus, the cetacean sequences summarized in Table 1 as well as *Artiodactyla *sequences were analyzed first classically, with gaps coded as missing characters, and secondly, with gaps coded as supplementary binary characters (see Fig. 5). For each analysis, two independent Bayesian searches were performed. The phylogenetic trees presented in Figure 4 result from a consensus of 20,000 trees sampled after standard deviation between the two runs dropped below 0.01. They show highly supported nodes. The phylogenetic analysis performed on the complete segment (Fig. 4a) confirmed the clustering by sex-chromosome copy in *Cetartiodactyla *(*Stenella cœruleoalba*, *Balænoptera physalus*, *Grampus griseus*, *Tursiops truncatus*, *Bos taurus *and *Ovis aries*) whereas *Amel-X *and *Amel-Y *clustered together in other mammals (*Homo sapiens*, *Sus scrofa*) together with *Amel-X *from *Cetartiodactyla*. On the other hand, phylogeny inferred without taking into account the insertion gave a different result (Fig. 4b): whereas haplotypes also clustered by chromosome in cetaceans, no signal related either to species history or to chromosome bearing could be seen in the other *Cetartiodactyla*. Hence, the phylogenetic signal related to species history seems to strengthen as we follow the tree from *Cetartiodactyla *towards primates. This partial, homoplasic, persistence of the phylogenetic signal may be explained by the influence of the region surrounding the insertion. This could be the result of the old age of the insertion (74–87 myrs [27]). The subsequent loss of homology may have given rise to a more divergent evolution between chromosomes in some taxa (*Cetacea*) than in others (*Artiodactyla*).

It would be interesting to study this region at the whole clade level by combining sequence and indel characters in the same analysis. This could give clues to test the many hypotheses about basal radiation of *Cetartiodactyla *(e.g. [5,6,8]). Given the presumably basal position of the *Suioidea *and *Tylopoda *in the *Cetartiodactyla *phylogeny ([7] and Fig. 3), we hypothesize that the major evolutionary event represented by the insertion (illustrated by an arrow Figure 4a) occurred once in the *Cetacea*-*Ruminantia *clade and not in the remaining *Cetartiodactyla*.

The presence of this large insertion in the *Amel-Y *copy can be useful for sex determination.

Evolutionary history also indicates that our sexing technique is applicable, in addition to cetaceans, to over a wide range of *Cetartiodactyla *species including domestic and wild species, in particular the widespread *Ruminantia *(*Bovidae*, *Capridae *and most likely *Cervidae*). It is however not suitable to Suiformes and further studies are required to confirm that the technique is also not applicable to Camelidae, given their even more basal position in the *Cetartiodactyla *phylogeny.

### Use in pedigree assessment and population genetics

In dolphins, the *Amel-Y *fragments amplified with the SC1-SC2 primer pair were easily sequenced without the need of cloning since amplification was Y chromosome-specific. From the ten Striped dolphin samples sequenced, nine were males, and we could deduce seven distinct Y-haplotypes (one haplotype represented by three individuals and four individual haplotypes) bearing 64 polymorphic sites (nucleotide diversity π = 0.004 ± 0.0007). Half of these were in the ~460 bp insertion. An alignment of polymorphic sites is presented in Figure 5 (a). Strikingly, these sequences showed two highly divergent haplogroups, diverging by a mean of 49 substitutions. This concords with our results that support the probable existence of two subspecies within the Mediterranean sea (unpublished data). Moreover, one of these haplogroups displayed a high degree of polymorphism, with 24 informative sites, whereas the others showed only eight. These values are sufficient for use in pedigree analysis and population genetics, as the Y chromosome counterpart of the mitochondrial d-loop in this species. Indeed, in striped dolphin the intra-specific (inter-group) divergence is greater than inter-specific divergence with a mean of 45 nucleotide substitutions between the striped dolphin and fin whale sequences. There is an average of 0.048 ± 0.01 substitutions per site when comparing the two striped dolphin populations. This is comparable to the divergence observed between each population and the Common dolphin (0.058 ± 0.03) and confirms that nucleotide diversity is one order of magnitude higher than the range observed (10^-4^) for Y chromosome markers in mammals [12]. As for the mitochondrial d-loop, the size of the amplified fragment slightly limits the use of the technique. Some degraded samples do not amplify; even so, a particularly degraded sperm whale sample was still amplifiable (data not shown).

Since the Y chromosome population is expected to have a small effective size, it is more likely to be affected by genetic drift. Thus, it reflects more recent demographic events such as bottlenecks, expansions or founder effects [28]. To study this sort of event, one needs a marker whose diversity is high enough to allow the reconstruction of gene genealogies with the least ambiguities and in regions where recombination does not interfere with the uniqueness of the trees. For this purpose, highly variable microsatellites represent valuable markers but they require intensive computing methods to take into account uncertainties in the trees arising from alleles that are identical by state and not by descent (homoplasies) [29,30]. Adding a new sequence marker is therefore of interest for Y-chromosome population genetics in *Cetartiodactyla*. Moreover, the Bayesian estimate of mutation rate on each edge of both trees in Fig. 4, jointly computed with phylogenetic inference, shows high values for a marker of nuclear DNA: between 10^-8 ^and 10^-10 ^substitutions per site and per year in all *Cetartiodactyla *branches. This value is intermediate between those of mitochondrial d-loop and nuclear DNA in mammals [31,32].

### Functional perspectives in amelogenin evolution

We found two stop codons at amino acid positions 98 and 99 of exon 5 in all Y chromosome copies of amelogenin in the four studied cetacean species (positions 988–993 of sequence EMBL:AM744959). The *Amel-Y *gene product may therefore be truncated in these species or represent a pseudogene as already observed in species from most of the other eutherian clades [33]

## Conclusion

The 460 bp insertion studied represents a single-event synapomorphy among most *Cetartiodactyla*. Together with the presence of other numerous indels informative at the order-level, it could help resolve the phylogenic discrepancies between hippopotamuses, pigs, camels and other *Cetartiodactyla *observed by many authors [7-9]. In addition, we demonstrate higher diversity within a single sequence than has yet been observed in multi-sequence assays [34]. This high diversity should allow the use of this sequence as the male counterpart of the mitochondrial control region. The applications would include inference of male-driven evolution in population genetics as applied to breeding management; domestication studies in archaeogenetics [35]; conservation biology (population history, sex-biased dispersal, admixture); or for testing sex-biased selection [28]. Amelogenin intron 4 amplification will also be an efficient tool for sexing *Ruminantia *and *Cetacea*. This will be useful for many fields of veterinary and forensic science (embryo technologies, *in vitro *fertilization, meat products). Finally, amelogenin amplification could also be a helpful tool for conservation biology through sampling of dead animals, faecal remains and biopsies of free-ranging animals like whales and dolphins. Amelogenin amplification in *Cetartiodactyla *therefore is a simple, single-step procedure with a wide range of applications.

## Methods

### Laboratory procedures

Biological material was isolated from soft tissues sampled from dead stranded cetaceans and extracted using a classical phenol-chloroform protocol [36]. We used heterologous primers, X5 (5'-GTGCTTACCCCTTTGAAGTG-3') and X6 (5'-CTTCCTCCCGCTTGGTCTTG-3'), designed from the amelogenin intron 4 and exon 5 of *Homo sapiens *X chromosome (GenBank:NC 000023, chrX:11221454–11228802) reference assembly Build 36.3, to amplify the homologous region in cetacean amelogenin. This region of *Amel-X *is 92% identical to *Amel-Y*.

We subsequently cloned and sequenced these PCR fragments and designed oligonucleotide primers specific to the *Artiodactyla*. They are anchored in exons 4 and 5 of *Amel-X *and *Amel-Y*, which allows complete amplification of the 4^th ^intron (SC1: 5'-CAAGCATGCATTTCAATTCCC-3' and SC2: 5'-CTGCATGGGGAACATCGGAG-3'). Optimal PCR conditions were adjusted by using temperature (49–62°C) and MgCl_2 _(1–2.5 mM) gradients. Following this optimization step, the PCR amplifications were conducted in a reaction mix consisting of 477 mM KCl, 1 mM Tris/HCl pH 8.3, 1.5 mM MgC1_2_, 250 μM each dNTP, 2 pmol/μl of each primer, 3 units of Taq polymerase in a final volume of 25 μl. Cycling was conducted as follows: 95°C for 2 min, followed by 30 cycles of denaturation at 94°C for 1 min, annealing at 55°C for 45 s, extension at 72°C for 1 min, and a final extension at 70°C for 10 min. PCR products were run on 1.2% agarose gel ethidium bromide stained, alongside a 1 kbp ladder (New England Biolabs, County Road, MA). In order to validate the assay, the gender, if identified during examination of the stranded carcasses, was recorded (22 out of 38 samples). These amplifications were conducted in eight Cetacean species: 20 Striped dolphin (*Stenella cœruleoalba*), five Fin whales (*Balaenoptera physalus*), four Bottlenosed dolphins (*Tursiops truncatus*), three Common dolphins (*Delphinus delphis*), three Gray whales (*Eschrichtius robustus*), two Sperm whales (*Physeter macrocephalus*), one Minke whale (*Balænoptera acutorostrata*) and one Risso's dolphin (*Grampus griseus*).

Of these, we sequenced striped dolphins (9 males and one female), fin whales (two males and two females), common dolphin (2 males), gray whale (one female) and Risso's dolphin (one female). Sequencing was performed on an ABI Prism sequencer (Applied Biosystems, Foster City, CA) with the dye terminator protocol directly for fragments amplified using the same (SC1-SC2) primer pair or after cloning in pGEM-T (Promega, Madison, WI) for fragments amplified using the X5-X6 pair. Each sample was sequenced from at least two independent amplifications for greater reliability.

### Bioinformatic analyses

The obtained sequences were aligned using ClustalW, a clustering-based sequence alignment algorithm [37], with bottlenosed dolphin (GenBank:AY787743S2), cow (GenBank:AB091789–AB091790), pig (GenBank:AF328419 and AB091792), sheep (GenBank:AY604731) and human (GenBank:NT_011757 from 9098117 to 9098612 and GenBank:NC_000024 from 6796200 to 6796719) sequences. Striped dolphin haplotypes were named according to membership in one of the two Mediterranean populations inferred by population genetic data on mitochondrial and microsatellite DNA (data not shown). *Stenella cœruleoalba YAn *thus represents the n^th ^haplotype from population 1 while *Stenella cœruleoalba YBn *represents the n^th ^haplotype from population 2.

Phylogenic inference was performed using a Bayesian Monte Carlo Markov Chain (MCMC) approach, implemented in Mrbayes [38], that allows determining posterior probability of each node of the tree.

Given the high indel density in this region, data were divided into two partitions: a sequence matrix (1120 characters corresponding to nucleotides) and a binary character matrix (presence/absence of the indels). Gap coding was achieved using Modified Complex Indel Coding [39] in which parsimony-informative gap characters were scored from pairwise unambiguously aligned sequences. Hence, a total of 47 gap characters were included (summarized in Figure 5b). We used a General Time Reversible (GTR) model, allowing for a proportion of invariant sites for sequence partition, and a binary F81-like model with a variable ascertainment bias for the insert's absence/presence (state 0/state 1) for the binary partition. In this model, π_0 _and π_1 _are the stationary probabilities of the two states and π_0_/π_1 _the rate of transition from state 1 to 0.

Patterns of genetic diversity were computed using DnaSP [40]. Specifically, the average number of substitutions was computed using Nei's equations 10.5, 10.6, 10.7 [41].

## Authors' contributions

MM performed the experiments, manuscript writing and editing.

BC was responsible for funding, supervision of the research project and manuscript writing.
